# Fruit, vegetable and vitamin C intakes and plasma vitamin C: cross‐sectional associations with insulin resistance and glycaemia in 9–10 year‐old children

**DOI:** 10.1111/dme.13006

**Published:** 2015-11-23

**Authors:** A. S. Donin, J. E. Dent, C. M. Nightingale, N. Sattar, C. G. Owen, A. R. Rudnicka, M. R. Perkin, A. M. Stephen, S. A. Jebb, D. G. Cook, P. H. Whincup

**Affiliations:** ^1^ Population Health Research InstituteSt George's, University of LondonLondonUK; ^2^ Institute of Cardiovascular and Medical SciencesUniversity of GlasgowGlasgowUK; ^3^ Medical Research Council Human Nutrition ResearchCambridgeUK; ^4^ Department of Nutritional SciencesUniversity of SurreyGuildfordUK; ^5^ Nuffield Department of Primary Care Health SciencesUniversity of OxfordOxfordUK

## Abstract

**Aim:**

To examine whether low circulating vitamin C concentrations and low fruit and vegetable intakes were associated with insulin resistance and other Type 2 diabetes risk markers in childhood.

**Methods:**

We conducted a cross‐sectional, school‐based study in 2025 UK children aged 9–10 years, predominantly of white European, South‐Asian and black African origin. A 24‐h dietary recall was used to assess fruit, vegetable and vitamin C intakes. Height, weight and fat mass were measured and a fasting blood sample collected to measure plasma vitamin C concentrations and Type 2 diabetes risk markers.

**Results:**

In analyses adjusting for confounding variables (including socio‐economic status), a one interquartile range higher plasma vitamin C concentration (30.9 μmol/l) was associated with a 9.6% (95% CI 6.5, 12.6%) lower homeostatic model assessment of insulin resistance value, 0.8% (95% CI 0.4, 1.2%) lower fasting glucose, 4.5% (95% CI 3.2, 5.9%) lower urate and 2.2% (95% CI 0.9, 3.4%) higher HDL cholesterol. HbA_1c_ concentration was 0.6% (95% CI 0.2, 1.0%) higher. Dietary fruit, vegetable and total vitamin C intakes were not associated with any Type 2 diabetes risk markers. Lower plasma vitamin C concentrations in South‐Asian and black African‐Caribbean children could partly explain their higher insulin resistance.

**Conclusions:**

Lower plasma vitamin C concentrations are associated with insulin resistance and could partly explain ethnic differences in insulin resistance. Experimental studies are needed to establish whether increasing plasma vitamin C can help prevent Type 2 diabetes at an early stage.

## What's new?

- We examined the cross‐sectional associations between intakes of fruit, vegetables and vitamin C, circulating vitamin C concentrations and insulin resistance in UK children.
- Circulating vitamin C concentration was inversely associated with insulin resistance, while intakes of fruit, vegetable and vitamin C were unrelated to insulin resistance.
- Low levels of circulating vitamin C concentrations in UK South‐Asian children could contribute to their higher insulin resistance.
- Further studies (including trials) are needed to examine the association of vitamin C with insulin resistance and its potential implications for Type 2 diabetes prevention.

**What's new?**

- We examined the cross‐sectional associations between intakes of fruit, vegetables and vitamin C, circulating vitamin C concentrations and insulin resistance in UK children.
- Circulating vitamin C concentration was inversely associated with insulin resistance, while intakes of fruit, vegetable and vitamin C were unrelated to insulin resistance.
- Low levels of circulating vitamin C concentrations in UK South‐Asian children could contribute to their higher insulin resistance.
- Further studies (including trials) are needed to examine the association of vitamin C with insulin resistance and its potential implications for Type 2 diabetes prevention.

## Introduction

Type 2 diabetes is a major public health problem, both in the UK and globally, affecting increasingly young age groups 1. Diet and nutrition are strongly implicated in the aetiology of Type 2 diabetes 2, although the specific causal factors, other than high energy intake leading to excess weight, remain unknown 3. Earlier studies in adults have suggested that low intakes of fruit, vegetables and vitamin C could be important determinants of Type 2 diabetes risk 4; however, more recent reports have suggested that the associations between fruit and vegetables and Type 2 diabetes risk are weak, with low fruit consumption associated with at most a 10% increase in Type 2 diabetes risk 5, 6. Furthermore, recent studies of the relationships between vitamin C intake and Type 2 diabetes risk have failed to confirm the earlier association 7. One report has suggested, however, that low circulating vitamin C concentration (a more direct marker of vitamin C status than intake) is prospectively associated with increased Type 2 diabetes risk 8. This association, if causal, could be important not only in explaining individual risk of Type 2 diabetes but also ethnic differences in risk; UK South Asians have low circulating vitamin C concentrations 9 and are at high risk of Type 2 diabetes 10.

Type 2 diabetes is increasingly occurring in childhood and adolescence 1 and is now recognized to have its origins in early life; however, there is little information on the dietary determinants of early Type 2 diabetes risk in childhood, and specifically on the associations between fruit, vegetable and dietary vitamin C intakes, plasma vitamin C concentrations and Type 2 diabetes risk markers (particularly insulin resistance and glycaemia) in childhood. We therefore investigated these associations in UK children aged 9–10 years in the Child Heart and Health Study in England (CHASE). We also examined the potential contribution of these dietary and nutritional factors to emerging ethnic differences in insulin resistance.

## Research design and methods

The CHASE study examined markers of Type 2 diabetes risk and their determinants in a multi‐ethnic population of children aged 9–10 years. The methods have been described in detail elsewhere 11. Balanced numbers of children of South‐Asian, black African‐Caribbean and white European origin were invited to take part, drawn from a stratified random sample of 200 primary schools in London, Birmingham and Leicester. Ethical approval was provided by the relevant Multicentre Research Ethics Committee and parents or guardians provided informed written consent. Participating children completed questionnaires, had physical measurements and provided a fasting blood sample. The present study was based on data collected in the last 85 schools (visited between February 2006 and February 2007), in which detailed information on diet was collected using a 24‐h recall assessment, and physical activity was objectively assessed using accelerometry.

### Physical and blood measurements

Three trained observers took height and weight measurements. Bioelectrical impedance was measured using a Bodystat 1500 body composition analyser (Bodystat Ltd, Isle of Man, UK) and was used to derive fat‐free mass and fat mass using validated ethnic‐specific equations 12; fat mass was presented as a height‐standardized index [fat mass (kg)/height (m)^5^]. Children provided blood samples after an overnight fast which were analysed by investigators blind to participants' ethnicity. Assay methods for insulin, glucose, HbA_1c_, urate and blood lipids have been described elsewhere 11, 13. Vitamin C was measured in heparinized plasma which was separated within 2 h of collection, treated with metaphosphoric acid immediately after separation and then snap‐frozen in dry ice and stored in darkness at −70°C until analysed within 3 months of collection using a fluorometric method at the MRC Human Nutrition Research Centre 14. Estimates of between‐batch imprecision were 11.5, 7.9 and 7.6% at plasma vitamin C concentrations of 31, 84 and 136 μmol/l, respectively. Children provided a sample of saliva, which was measured with a gas‐liquid chromatography method (detection limit 0.1 ng/ml) to determine levels of cotinine.

### Ethnicity and socio‐economic status

Ethnicity of the child was categorized using self‐defined ethnicity of both parents or by using parental information on the ethnicity of the child. In a small number of participants for whom this information was not available (1%), child‐defined place of origin of parents and grandparents was used, cross‐checked with the observer‐defined ethnic appearance of the child. Children were broadly classified into four main ethnic groups (‘white European’, ‘black African‐Caribbean’, ‘South Asian’ or ‘other’), with a more detailed classification into 10 ethnic subgroups (white European, black African, black Caribbean, black other, Indian, Pakistani, Bangladeshi, South‐Asian other, Asian other, other) as previously described 11. Both parents and children provided information on parents' occupation, which was coded using the National Statistics Socioeconomic Classification 15, resulting in the following classifications: managerial/professional; intermediate; routine/manual; and economically inactive, as previously described 16.

### Detailed assessment of diet and physical activity and other factors

Children were interviewed by a research nutritionist who conducted a single, structured 24‐h recall of the foods eaten on the previous day 17, which included key elements of the US Department of Agriculture multiple pass method 18. Full details have been previously described 19. Memory cues were used to aid recall, and photographs of common foods were used to help the child estimate portion sizes. Food and nutrient intakes were calculated by the Medical Research Council Human Nutrition Research centre using an in‐house food composition database 20. Children were asked to wear an Actigraph GT1M accelerometer for a 7‐day period immediately after the survey; further details of these measurements have been published elsewhere 21. Fruit and vegetable intakes were calculated as the total weight of these foods consumed, and included, where possible, those consumed as part of composite dishes. All fruit and vegetables, including fresh, frozen, canned and dried, were included. Potatoes were not included as a vegetable. No drinks were included in the analyses of fruit and vegetables. Both children and parents were asked about any specific health problems experienced by the child and about regular vitamin supplement use. Parents were asked about their own and parental history of diabetes.

### Statistical methods

Statistical analyses were carried out in stata (version 12.1; StataCorp LP, College Station, TX, USA). All main outcome variables were log‐transformed and regression coefficients were presented as percentage changes. Multilevel linear regression models were fitted to provide adjusted means and adjusted differences in risk factors and their 95% CIs per one interquartile range increase in each dietary or nutritional variable, to ensure the comparability of different exposures. All analyses were adjusted for sex, age (in quartiles), total energy intake and ethnicity (10 groups) and month of assessment (11 groups, to allow for seasonal differences in nutritional markers). In addition, school was fitted as a random effect to take account of the natural clustering of children within school. In further analyses to take other potential confounders into account, fat mass index, salivary cotinine concentrations, physical activity (counts per min), blood pressure (all fitted as continuous variables) and socio‐economic status (categorical, four‐level variable) were also included in the multilevel linear regression models.

## Results

Of the 3679 children invited, 2529 (69%) took part in the present study, of whom 2338 children (92%) provided fasting blood samples. One child with Type 1 diabetes was excluded. Among the remainder, 2025 children also completed 24‐h dietary recalls and were therefore included in the present analyses; participants had a mean age of 10.0 years (95% reference range 9.3 to 10.6 years) and 53% were girls. Participation rates were slightly higher among white Europeans (71%) and South Asians (73%) than among black African‐Caribbean (65%) and other ethnic groups (70%). The numbers of children studied in each ethnic group were similar (*n* = 504, 523, 502, and 496 respectively). The representation of parental socio‐economic position included 27% in managerial/professional occupations, 26% in intermediate and 33% in routine/manual, with 9% economically inactive and 5% unclassified.

Physical and demographic characteristics, blood analytes and fruit, vegetable and vitamin C intakes are summarized by ethnicity in Table 1 and by gender in Table S1. There were marked ethnic differences in Type 2 diabetes risk markers, consistent with those previously reported in the whole CHASE population 11 and in plasma vitamin C concentrations, highest in white Europeans and lowest in South Asians, particularly Bangladeshis. There were also ethnic differences in vegetable intakes: black African children had the highest intakes and white European children the lowest intakes. Dietary vitamin C intakes tended to be higher in white Europeans and lower in South Asians (as for plasma vitamin C), although these differences were not statistically significant. Vitamin supplement use was highest in the black Caribbean children and lowest in the South‐Asian children. The lowest proportion of children in the highest socio‐economic status group was in Bangladeshis and the highest in black Caribbeans. Girls had significantly higher levels of adiposity (fat mass index), insulin, homeostatic model assessment of insulin resistance values, triglyceride and C‐reactive protein. Boys had higher levels of glucose, systolic blood pressure and HDL cholesterol. Vegetable intake was higher in girls, while fruit intake, vitamin C intake and plasma vitamin C concentrations showed no marked sex differences. Intercorrelations of nutritional markers among all children are summarized in Table S2 and then stratified by vitamin supplement use. Among all children, modest correlations were observed between vitamin C intake and plasma vitamin C level (*r* = 0.26), between fruit intake and vitamin C intake (*r* = 0.32) and between fruit intake and plasma vitamin C (*r* = 0.11); associations for vegetable intake were weaker. In stratified analyses, these correlations tended to be stronger among children who were not taking supplements than among those who were.

**Table 1:** Demographic data, Type 2 diabetes risk markers, dietary fruit, vegetable and vitamin C intakes and plasma vitamin C, stratified by ethnic group

	White Europeans (*n* = 504)	Black Caribbeans (*n* = 185)	Black Africans (*n* = 278)	Indians (*n* = 127)	Pakistanis (*n* = 200)	Bangladeshis (*n* = 164)	Other (*n* = 567)	*P* (no difference)
	Mean (sd/IQR)	Mean (sd/IQR)	Mean (sd/IQR)	Mean (sd/IQR)	Mean (sd/IQR)	Mean (sd/IQR)	Mean (sd/IQR)	
Age, years	10.0 (0.4)	9.9 (0.4)	9.9 (0.4)	10.0 (0.4)	10.0 (0.3)	10.0 (0.4)	10.0 (0.4)	0.87
Insulin†, mU/l	6.0 (4.2)	7.5 (5.2)	7.0 (5.8)	8.4 (6.7)	7.6 (5.9)	9.1 (7.0)	6.9 (5.6)	< 0.0001
Insulin resistance†, HOMA	0.7 (0.6)	0.9 (0.8)	0.9 (0.7)	1.0 (0.8)	1.0 (0.8)	1.1 (0.9)	0.9 (0.7)	< 0.0001
HbA_1c_, mmol/mol	33	34	34	36	34	34		< 0.0001
HbA_1c_ †, %	5.2 (0.3)	5.3 (0.5)	5.3 (0.4)	5.4 (0.4)	5.3 (0.4)	5.3 (0.4)	5.2 (0.4)	< 0.0001
Glucose†, mmol/l	4.5 (0.5)	4.4 (0.4)	4.4 (0.4)	4.5 (0.4)	4.5 (0.5)	4.6 (0.4)	4.5 (0.5)	0.01
Triglyceride†, mmol/l	0.8 (0.4)	0.8 (0.4)	0.7 (0.3)	1.0 (0.5)	0.9 (0.4)	1.0 (0.6)	0.8 (0.5)	< 0.0001
HDL cholesterol, mmol/l	1.6 (0.3)	1.6 (0.3)	1.6 (0.3)	1.5 (0.4)	1.5 (0.3)	1.4 (0.3)	1.5 (0.4)	0.003
C‐reactive protein†, mg/l	0.4 (0.7)	0.5 (1.3)	0.5 (1.0)	0.6 (1.3)	0.8 (1.4)	0.5 (0.9)	0.5 (1.0)	< 0.0001
Urate†, mmol/l	0.2 (0.1)	0.2 (0.1)	0.2 (0.1)	0.2 (0.1)	0.2 (0.1)	0.2 (0.1)	0.2 (0.1)	< 0.0001
Systolic BP, mmHg	104.5 (10.5)	104.8 (11.2)	103.5 (10.6)	104.2 (11.4)	103.8 (10.6)	103.5 (11.7)	105.2 (10.6)	0.62
Diastolic BP, mmHg	62.2 (8.7)	62.9 (9.5	63.0 (9.6)	64.0 (10.0)	62.8 (9.2)	62.9 (9.8)	62.6 (9.7)	< 0.0001
Fat mass index†, kg/m^5^	2.1 (1.1)	1.9 (1.2)	1.9 (1.0)	2.1 (1.3)	2.1 (1.1)	2.2 (1.1)	2.1 (1.2)	< 0.0001
Dietary vitamin C†, mg	87.2 (95.3)	82.3 (78.3)	86.9 (104.8)	88.2 (95.9)	77.9 (89.1)	66.7 (83.8)	81.5 (97.8)	0.26
Fruit intake†, g	136.9 (120.0)	121.0 (100.0)	140.2 (105.0)	124.5 (120.0)	125.4 (100.0)	126.2 (100.0)	121.8 (104.0)	0.29
Vegetable intake†, g	70.4 (78.8)	77.6 (105.0)	89.9 (93.0)	77.3 (117.5)	78.0 (69.8)	76.7 (103.2)	76.2 (80.5)	< 0.0001
Plasma vitamin C, μmol/l	89.1 (23.3)	86.8 (19.8)	82.8 (22.5)	80.8 (23.4)	78.1 (23.3)	65.8 (24.6)	85.5 (21.4)	< 0.0001
Energy intake, kcals	1,813 (484)	1,815 (484)	1,839 (520)	1,864 (476)	1,952 (528)	1,954 (556)	1,814 (460)	0.019
Cotinine, ng/ml	1.1 (2.0)	0.4 (0.7)	0.2 (0.4)	0.4 (1.8)	0.4 (0.8)	1.4 (10.5)	0.8 (1.6)	0.002
Physical activity, counts p/min	493.8 (102.6)	494.7 (105.3)	485.5 (103.5)	427.2 (116.2)	468.4 (109.5)	448.3 (105.0)	506.2 (104.7)	< 0.0001
Sex, % girls	51	46	59	47	53	58	49	0.11‡
Vitamin supplement users, %	19	24	10	17	12	5	23	< 0.0001‡
Parental socio‐economic status,	32	36	27	28	16	11	29	< 0.0001‡
% managerial/professional								

BP, blood pressure; HOMA, homeostatic model assessment; IQR, interquartile range.

†Geometric means and IQRs are given for log‐transformed variables. Means and geometric means, sd and IQR are based on raw data. *P* values are adjusted for age, sex, month school as a random effect.

‡*P* values for frequencies are derived from chi‐squared tests.

### Associations between fruit, vegetable, vitamin C intakes, plasma vitamin C and Type 2 diabetes risk markers

Table 2 shows the differences in Type 2 diabetes risk markers per interquartile range increase in intakes of fruit, vegetables and vitamin C and plasma vitamin C concentrations, in analyses adjusted for age, sex, month, total energy intake, ethnicity and school (random effect). No associations were found between fruit, vegetable or dietary vitamin C intake and Type 2 diabetes risk markers. Plasma vitamin C concentrations, in contrast, showed strong inverse associations with fasting insulin and insulin resistance, glucose and urate, and a positive association with HDL cholesterol. Unexpectedly, a positive association was also found for HbA_1c_ levels. The inverse associations between plasma vitamin C and insulin resistance showed a clearly graded pattern (Figure S1). Table S3 presents additional information on the strengths of these associations, including the percentage differences in Type 2 diabetes risk markers for a one interquartile range increase in plasma vitamin C and percentage differences in Type 2 diabetes risk markers between the highest and lowest quartile of plasma vitamin C. It also presents the absolute differences in Type 2 diabetes risk markers for a one interquartile range increase in plasma vitamin C.

**Table 2:** Percentage differences in Type 2 diabetes risk markers per one interquartile range increase in dietary fruit, vegetables and vitamin C intakes and plasma vitamin C concentrations in 2025 children

Outcome	All fruit (g)		All vegetables (g)		Dietary vitamin C (mg)		Plasma vitamin C (μmol/L)	
	% difference (95% CI)	*P*	% difference (95% CI)	*P*	% difference (95% CI)	*P*	% difference (95% CI)	*P*
Insulin (mU/l)	0.7 (−3.2, 4.8)	0.73	0.8 (−2.7, 4.4)	0.65	1.0 (−2.3, 4.4)	0.54	−11.5 (−14.7, −8.1)	< 0.0001
Insulin resistance (HOMA)	0.7 (−3.2, 4.7)	0.73	1.0 (−2.5, 4.6)	0.58	0.9 (−2.4, 4.3)	0.59	−11.7 (−14.9, −8.3)	< 0.0001
HbA_1c_ (%)	0.2 (−0.2, 0.6)	0.40	0.2 (−0.1, 0.6)	0.19	0.2 (−0.1, 0.5)	0.24	0.5 (0.1, 0.9)	0.01
Glucose (mmol/l)	−0.1 (−0.6, 0.3)	0.59	0.2 (−0.2, 0.6)	0.33	−0.1 (−0.5, 0.3)	0.51	−0.8 (−1.2, −0.4)	0.0002
Triglycerides (mmol/l)	0.5 (−1.9, 3.0)	0.67	−2.0 (−4.1, 0.1)	0.07	1.1 (−1.0, 3.2)	0.30	−0.2 (−2.5, 2.1)	0.84
HDL cholesterol (mmol/l)	−0.1 (−1.4, 1.3)	0.92	0.5 (−0.7, 1.7)	0.38	1.0 (−0.1, 2.2)	0.08	2.9 (1.6, 4.2)	< 0.0001
C‐reactive protein (mg/l)	−1.3 (−9.2, 7.2)	0.75	−1.6 (−8.7, 6.0)	0.66	4.4 (−2.6, 11.9)	0.23	−7.8 (−14.7, −0.3)	0.04
Urate (mmol/l)	1.2 (−0.3, 2.8)	0.12	0.1 (−1.3, 1.5)	0.92	−0.3 (−1.6, 1.0)	0.66	−4.9 (−6.3, −3.5)	< 0.0001
Fat mass index	0.0 (−2.3, 2.3)	0.98	−1.8 (−3.8, 0.2)	0.08	0.7 (−1.2, 2.7)	0.47	−2.1 (−4.3, 0.0)	0.05

HOMA, homeostatic model assessment; IQR, interquartile range.

Percentage differences are adjusted for age (quartiles), sex, month, total energy intake (kcals), ethnic group and school (random effect). A one IQR increase represents 170 g/day for fruit intake, 100 g/day for vegetable intake, 95.3 mg/day for vitamin C intake and 30.9 μmol/l for plasma vitamin C concentration.

Associations between plasma vitamin C and a range of potential confounding factors are summarized in Table S4. Plasma vitamin C concentration showed no associations with age, sex, objectively measured physical activity, adiposity, total energy intake or cotinine concentration, but differed markedly by socio‐economic position (with markedly higher vitamin C concentrations among children with parents in managerial/professional occupations), by ethnic group and by vitamin supplement use. The associations between plasma vitamin C and Type 2 diabetes risk markers, however, were little affected by additional adjustments for adiposity, socio‐economic position, physical activity and blood pressure (Table 3). After adjustment for these factors, the strengths of associations between plasma vitamin C concentrations and fasting insulin, insulin resistance, fasting glucose, urate, and HDL cholesterol were all reduced by less than a quarter, while the association between plasma vitamin C and HbA_1c_ became slightly stronger after adjustment. The associations between plasma vitamin C and Type 2 diabetes risk markers did not differ markedly between white Europeans, South Asians and black African‐Caribbeans (data not presented). In further sensitivity analyses, the associations between plasma vitamin C and Type 2 diabetes risk markers were not affected by adjustment for a family history of diabetes (data not presented). The exclusion of 337 children reported to be taking dietary supplements and the exclusion of 22 children who were potentially active smokers, with salivary cotinine levels > 12 ng/ml 22, did not materially affect the associations between plasma vitamin C concentration and Type 2 diabetes risk markers (Table S5). The exclusion of small numbers of children with plasma vitamin C levels associated with clinical (< 11.4 μmol/l) or moderate (11.5–20 μmol/l) vitamin C deficiency (*n* = 3 and *n* = 7, respectively) also had no material effect on the associations (data not presented).

**Table 3:** Percentage differences in Type 2 diabetes risk markers per one interquartile range increase in circulating plasma vitamin C concentrations: effect of adjustment for potential confounders

Outcome	Model	Difference per 1 IQR increase in plasma vitamin C	
		% difference (95% CI)	*P*
Insulin (mU/L)	Model 1	−11.5 (−14.7, −8.1)	< 0.0001
	Model 2	−9.6 (−12.6, −6.5)	< 0.0001
	Model 3	−9.5 (−12.5, −6.4)	< 0.0001
	Model 4	−7.7 (−11.1, −4.2)	< 0.0001
	Model 5	−7.2 (−10.6, −3.8)	< 0.0001
Insulin resistance (HOMA)	Model 1	−11.7 (−14.9, −8.3)	< 0.0001
	Model 2	−9.8 (−12.8, −6.7)	< 0.0001
	Model 3	−9.7 (−12.7, −6.6)	< 0.0001
	Model 4	−7.8 (−11.2, −4.3)	< 0.0001
	Model 5	−7.4 (−10.7, −4.0)	< 0.0001
HbA_1c_ (%)	Model 1	0.5 (0.1, 0.9)	0.01
	Model 2	0.6 (0.2, 1.0)	0.002
	Model 3	0.6 (0.2, 1.0)	0.003
	Model 4	0.7 (0.3, 1.1)	0.002
	Model 5	0.7 (0.3, 1.1)	0.001
Glucose (mmol/l)	Model 1	−0.8 (−1.2, −0.4)	0.0002
	Model 2	−0.8 (−1.2, −0.4)	0.0004
	Model 3	−0.8 (−1.2, −0.4)	0.0003
	Model 4	−0.6 (−1.1, −0.2)	0.01
	Model 5	−0.6 (−1.1, −0.1)	0.01
HDL cholesterol (mmol/l)	Model 1	2.9 (1.6, 4.2)	< 0.0001
	Model 2	2.4 (1.1, 3.6)	0.0002
	Model 3	2.2 (0.9, 3.4)	0.0005
	Model 4	2.0 (0.6, 3.4)	0.004
	Model 5	2.0 (0.6, 3.4)	0.004
Urate (mmol/l)	Model 1	−4.9 (−6.3, −3.5)	< 0.0001
	Model 2	−4.4 (−5.7, −3.0)	< 0.0001
	Model 3	−4.4 (−5.7, −3.1)	< 0.0001
	Model 4	−4.2 (−5.7, −2.7)	< 0.0001
	Model 5	−4.2 (−5.7, −2.6)	< 0.0001

HOMA, homeostatic model assessment; IQR, interquartile range.

Model 1: Percentage differences are adjusted for age (quartiles), sex, month, total energy intake, ethnic group and school (random effect).

Model 2: Adjusted as for model 1 plus adiposity (fat mass index).

Model 3: Adjusted as for model 2 plus socio‐economic status.

Model 4: Adjusted as for model 3 plus physical activity.

Model 5: Adjusted as for model 4 plus blood pressure.

Model 1, 2 and 3 includes 2025 children; model 4 and 5 includes 1539 children (486 children did not provide physical activity data). One IQR increase represents 30.9 μmol/l in plasma vitamin C concentrations.

### Ethnic differences in fruit, vegetable and dietary vitamin C intakes and plasma vitamin C concentrations: contributions to ethnic differences in insulin resistance

The effect on ethnic differences in fasting insulin and insulin resistance (based on comparisons with white Europeans) of adjusting for dietary intakes of fruit, vegetables and vitamin C and plasma vitamin C concentrations were examined (Table 4). South Asians (especially Bangladeshis), and to a lesser extent black Africans and black Caribbeans, had higher insulin and insulin resistance than white European children (as previously described in this study population 11). These ethnic differences in fasting insulin and insulin resistance were appreciably reduced by adjustment for plasma vitamin C in all ethnic groups; the effects were particularly marked in Pakistani and Bangladeshi children, in whom the differences were reduced by 21 and 23%, respectively. In contrast, adjustment for dietary fruit, vegetable and vitamin C intakes made no material difference to the observed ethnic differences in insulin resistance.

**Table 4:** Ethnic differences in insulin and insulin resistance (ethnic groups minus white Europeans): effect of additional adjustments for dietary and plasma vitamin C

Outcome	Adjustments	Black Caribbean differences (*n* = 185)	Black African differences (*n* = 278)	Indian differences (*n* = 127)	Pakistani differences (*n* = 200)	Bangladeshi differences (*n* = 164)
		% difference (95% CI)	% difference (95% CI)	% difference (95% CI)	% difference (95% CI)	% difference (95% CI)
Insulin (mU/L)	Standard†	29.3 (17.8, 41.9)***	22.2 (12.7, 32.5)***	39.0 (24.3, 55.5)***	20.5 (9.3, 32.9)**	40.8 (26.6, 56.5)***
	+ dietary vitamin C	29.3 (17.8, 41.9)***	22.2 (12.7, 32.5)***	39.1 (24.3, 55.5)***	20.6 (9.4, 33.0)**	41.0 (26.8, 56.8)***
	+ fruit intake	29.3 (17.8, 41.9)***	22.2 (12.6, 32.5)***	39.0 (24.2, 55.5)***	20.5 (9.3, 32.9)**	40.8 (26.6, 56.7)***
	+ vegetable intake	28.8 (17.4, 41.4)***	21.3 (11.8, 31.6)***	37.8 (23.1, 54.2)***	19.9 (8.7, 32.3)**	39.7 (25.5, 55.5)***
	+ plasma vitamin C	27.7 (16.4, 40.0)***	19.1 (9.9, 29.1)***	34.9 (20.7, 50.8)***	16.2 (5.4, 28.1)**	31.4 (18.0, 46.3)***
Insulin resistance (HOMA)	Standard†	29.7 (18.2, 42.3)***	21.9 (12.5, 32.2)***	39.0 (24.3, 55.5)***	21.0 (9.7, 33.3)**	40.8 (26.7, 56.5)***
	+ dietary vitamin C	29.7 (18.2, 42.3)***	21.9 (12.5, 32.2)***	39.1 (24.4, 55.6)***	21.1 (9.8, 33.5)**	41.0 (26.8, 56.7)***
	+ fruit intake	29.7 (18.2, 42.4)***	21.9 (12.4, 32.2)***	39.0 (24.3, 55.5)***	21.0 (9.7, 33.4)**	40.9 (26.7, 56.7)***
	+ vegetable intake	29.2 (17.7, 41.8)***	21.0 (11.6, 31.3)***	37.7 (23.1, 54.1)***	20.3 (9.1, 32.7)**	39.7 (25.6, 55.4)***
	+ plasma vitamin C	28.0 (16.8, 40.4)***	18.8 (9.6, 28.8)***	34.9 (20.7, 50.8)***	16.5 (5.7, 28.5)**	31.1 (17.8, 46.0)***

HOMA, homeostatic model assessment.

†Standard adjustments are made for age (quartiles), sex, month, adiposity (fat mass index), total energy intake and school (random effect) in all models; each nutritional factor was added separately in analysis. % reduction in coefficient.

** Significantly different from white Europeans at *P* < 0.001, *** significantly different from white Europeans at *P* < 0.0001.

## Discussion

In the present report, we describe associations between fruit, vegetable and vitamin C intakes, plasma vitamin C concentration and Type 2 diabetes risk markers in childhood. Lower plasma vitamin C concentrations were associated with higher levels of insulin resistance and there were consistent associations for HDL cholesterol, urate and fasting glucose (though not HbA_1c_) in UK children. Adjustment for the lower concentrations of plasma vitamin C among South‐Asian and black African‐Caribbean children accounted for part of the higher insulin resistance in these groups. No associations were found between dietary intake of fruits, vegetables or vitamin C and Type 2 diabetes risk markers.

The mean daily intakes of fruit and vitamin C in the present study were similar to those reported in the UK National Diet and Nutrition Surveys between 2008 and 2011 23, although vegetable intakes in the present study were slightly lower. The mean plasma vitamin C concentrations were higher than those in several other surveys, including the UK National Diet and Nutrition Surveys 23. These higher concentrations may well reflect the more rapid separation and snap‐freezing of blood samples for measurement of this unstable analyte in the present study than was possible in the national surveys.

In the present study, plasma vitamin C level had an inverse association with insulin resistance, while intakes of fruit, vegetables and vitamin C had no association. These novel findings in children are broadly consistent with the few studies in adults. In particular, the inverse association between plasma vitamin C concentrations and insulin resistance in the present study is consistent with a previous report, the EPIC‐Norfolk Study, which suggested that circulating vitamin C concentrations, to a greater extent than fruit and vegetable intake, was prospectively inversely associated with Type 2 diabetes risk 8, independently of potential confounders. These findings are also consistent with earlier reports in adults, suggesting that vitamin C supplementation is associated with reduced Type 2 diabetes risk 24. The reason for the anomalous positive association between plasma vitamin C and HbA_1c_ in the present study remains unclear, but could reflect an effect of circulating vitamin C levels on glycation of haemoglobin, as previously reported 25. The ethnic differences in plasma vitamin C reported in the present study are consistent with a previous study in adults, which showed that South‐Asian adults had lower plasma vitamin C than white Europeans, a difference which was not explained by differences in vitamin supplement use 9. To our knowledge, however, previous studies have not attempted to quantify the potential contribution of circulating vitamin C levels to ethnic differences in risks of insulin resistance or Type 2 diabetes. Our results are also consistent with recent prospective studies and systematic reviews of prospective studies in adults in finding no consistent evidence for associations between overall fruit and vegetable intakes and Type 2 diabetes risk 3, 5, 6 or for associations between vitamin C intake and Type 2 diabetes risk 7.

The study population was large and drawn from three major UK cities, with school‐based sampling designed to provide strong and balanced ethnic minority representation. The study included relevant early risk markers for Type 2 diabetes; insulin resistance was assessed using the homeostatic model assessment method which has been validated in children and provides estimates very similar to those of fasting insulin 26. HbA_1c_ provides an assessment of ambient glucose concentrations over ~3 months. Assessment of body fat was based on fat mass index derived from bioelectrical impedance, a more valid indicator of body fat than BMI, especially in this multi‐ethnic population 12. The assessment of fruit, vegetable and vitamin C intake was based on a single 24‐h diet recall, a practical method for large‐scale use that provides estimates of nutrient intakes which are valid though imprecise in this age group. The nutrient intakes reported in the present study are consistent with estimates in the National Diet and Nutrition Survey, which used a more detailed method of dietary data collection (7‐day weighed food diary) 27. The assessment of plasma vitamin C is based on a single measurement, influenced by vitamin C intake over the preceding week or so 28. The impacts of potentially important confounders of the vitamin C–insulin resistance association (particularly socio‐economic position and physical activity) were specifically examined and did not materially affect the results. The prevalence of cigarette smoking, another potentially important confounder 29, was very low (~1%); exclusion of active smokers was carried out and also had no material effect. Although the cross‐sectional study design limits the strength of evidence supporting a potentially causal association between plasma vitamin C and emerging Type 2 diabetes risk, this design is nevertheless particularly appropriate for examining short‐term associations between nutritional status and Type 2 diabetes risk markers.

Previous investigations of the associations between circulating vitamin C levels and chronic disease risk have drawn attention to the possibility of confounding, particularly by socio‐economic factors and health behaviours 30. In the present study, the association between plasma vitamin C and insulin resistance was moderately strong and graded, and appeared to be independent of adjustment for potential confounding factors (particularly socio‐economic status, physical activity and cigarette smoking). It also appeared consistent across different ethnic groups. No children in the study reported renal disease or other serious medical conditions, or treatment for high blood pressure or other serious medical conditions which could have influenced these associations; adjustment for family history of diabetes did not materially affect the results. These results raise the possibility of a causal association between circulating vitamin C levels and insulin resistance, although its nature and direction remain unclear. Although earlier reports noted that people with newly diagnosed Type 2 diabetes had low circulating vitamin C concentrations 31, subsequent investigations suggested that this reflected lower dietary vitamin C intakes rather than hyperglycaemia or insulin resistance, suggesting that diabetes and insulin resistance were unlikely to be primary causes of low vitamin C concentrations 32. Further evidence on the causal pathway underlying the vitamin C insulin resistance association is urgently needed.

The absence of detectable associations between intakes of fruit, vegetable and total vitamin C intake and insulin resistance, despite the presence of the association between plasma vitamin C level and insulin resistance could have several explanations. This contrasting pattern could reflect a stronger role for circulating vitamin C as a marker of systemic vitamin C status, more likely to be related to emerging Type 2 diabetes risk than vitamin C intake; however, the absence of associations could also reflect the fact that, although dietary vitamin C intake is an important determinant of plasma concentrations, it is difficult to assess precisely 28. Moreover, the influence of vitamin C intake on systemic vitamin C status is modified by other factors including absorption and bioavailability 28. These considerations would need to be taken into account in efforts to raise circulating vitamin C levels. Based on supplementary analyses in the present study, which are consistent with earlier reports 28, maintaining a higher plasma vitamin C concentration (~30 μmol/l, associated with an appreciably lower insulin resistance) would require a sustained increase in vitamin C intake in excess of 200 mg/day. Such an increase would require substantial and probably infeasible increases in fruit and vegetable consumption and would be more simply provided by vitamin C supplementation. Experimental testing of different strategies to increase circulating vitamin C levels, particularly in relevant ethnic minority populations would be needed to confirm this conclusion.

In conclusion, the strong inverse association between plasma vitamin C and insulin resistance in children raises the possibility that interventions to increase plasma vitamin C could be effective in early Type 2 diabetes prevention, perhaps especially in UK South‐Asian children. Further research, particularly using randomized controlled trials and Mendelian randomization studies, is needed to establish whether the circulating vitamin C–insulin resistance association is causal and whether it reflects the influence of other dietary components or wider biological or lifestyle factors.

## Funding sources

This work was supported by research grants from Diabetes UK (BDA 11/0004317**)** and the BUPA Foundation. Data collection in the CHASE Study was supported by grants from the Wellcome Trust (068362/Z/02/Z) and the National Prevention Research Initiative (NPRI) (G0501295). The Funding Partners for this NPRI award were: British Heart Foundation; Cancer Research UK; Department of Health; Diabetes UK; Economic and Social Research Council; Medical Research Council; Research and Development Office for the Northern Ireland Health and Social Services; Chief Scientist Office, Scottish Executive Health Department; and Welsh Assembly Government. Diabetes prevention research at St George's, University of London is supported by the NIHR CLAHRC South London. The views expressed are those of the author(s) and not necessarily those of the funding agencies, the NHS, the NIHR or the Department of Health.

## Competing interests

None declared.

## Supporting information


**Table S1** Blood markers, physical measurements, dietary intake and demographic information by gender
**Table S2** Correlations between fruit, vegetable, dietary vitamin C intakes and plasma vitamin C
**Table S3** Percentage and absolute differences in Type 2 diabetes risk markers for specific increases in plasma vitamin C concentrations
**Table S4** Population characteristics by quartiles of plasma vitamin C
**Table S5** Percentage differences in Type 2 diabetes risk markers per interquartile range increase in circulating plasma vitamin C concentrations: sensitivity analyses excluding children with high cotinine levels or who reported taking dietary supplements

**Figure S1** Fasting insulin by mean plasma vitamin C (μmol/l) in fourths.

## References

[1] International Diabetes Federation . IDF Diabetes Atlas, 6th ed Brussels, Belgium: International Diabetes Federation, 2013.

[2] Dyson PA , Kelly T , Deakin T , Duncan A , Frost G , Harrison Z *et al* Diabetes UK evidence‐based nutrition guidelines for the prevention and management of diabetes. Diabet Med 2011; 28: 1282–1288. 21699560 10.1111/j.1464-5491.2011.03371.x

[3] Carter P , Khunti K , Davies MJ . Dietary Recommendations for the Prevention of Type 2 diabetes: What Are They Based on? J Nutr Metab 2012; 2012: 847202. 22315675 10.1155/2012/847202 PMC3270422

[4] Boeing H , Weisgerber UM , Jeckel A , Rose HJ , Kroke A . Association between glycated hemoglobin and diet and other lifestyle factors in a nondiabetic population: cross‐sectional evaluation of data from the Potsdam cohort of the European Prospective Investigation into Cancer and Nutrition Study. Am J Clin Nutr 2000; 71: 1115–1122. 10799373 10.1093/ajcn/71.5.1115

[5] Cooper AJ , Sharp SJ , Lentjes MA , Luben RN , Khaw KT , Wareham NJ *et al* A prospective study of the association between quantity and variety of fruit and vegetable intake and incident type 2 diabetes. Diabetes Care 2012; 35: 1293–1300. 22474042 10.2337/dc11-2388 PMC3357245

[6] Muraki I , Imamura F , Manson JE , Hu FB , Willett WC , van Dam RM *et al* Fruit consumption and risk of type 2 diabetes: results from three prospective longitudinal cohort studies. BMJ 2013; 347: f5001. 23990623 10.1136/bmj.f5001 PMC3978819

[7] de Oliviera Otto MC , Alonso A , Lee DH , Delclos GL , Bertoni AG , Jiang R *et al* Dietary intakes of zinc and heme iron from red meat, but not from other sources, are associated with greater risk of metabolic syndrome and cardiovascular disease. J Nutr 2012; 142: 526–533. 22259193 10.3945/jn.111.149781 PMC3278268

[8] Harding AH , Wareham NJ , Bingham SA , Khaw K , Luben R , Welch A *et al* Plasma vitamin C level, fruit and vegetable consumption, and the risk of new‐onset type 2 diabetes mellitus: the European prospective investigation of cancer–Norfolk prospective study. Arch Intern Med 2008; 168: 1493–1499. 18663161 10.1001/archinte.168.14.1493

[9] Carter P , Gray LJ , Morris DH , Davies MJ , Khunti K . South Asian individuals at high risk of type 2 diabetes have lower plasma vitamin C levels than white Europeans. J Nutr Sci 2013; 2: 1–5. 10.1017/jns.2013.15 PMC4153325 25191570

[10] Tillin T , Hughes AD , Godsland IF , Whincup P , Forouhi NG , Welsh P *et al* Insulin resistance and truncal obesity as important determinants of the greater incidence of diabetes in Indian Asians and African Caribbeans compared with Europeans: the Southall And Brent REvisited (SABRE) cohort. Diabetes Care 2013; 36: 383–393. 22966089 10.2337/dc12-0544 PMC3554271

[11] Whincup PH , Nightingale CM , Owen CG , Rudnicka AR , Gibb I , McKay CM *et al* Early emergence of ethnic differences in type 2 diabetes precursors in the UK: the Child Heart and Health Study in England (CHASE Study). PLoS Med 2010; 7: e1000263. 20421924 10.1371/journal.pmed.1000263 PMC2857652

[12] Nightingale CM , Rudnicka AR , Owen CG , Donin AS , Newton SL , Furness CA *et al* Are ethnic and gender specific equations needed to derive fat free mass from bioelectrical impedance in children of South asian, black african‐Caribbean and white European origin? Results of the assessment of body composition in children study. PLoS One 2013; 8: e76426. 24204625 10.1371/journal.pone.0076426 PMC3799736

[13] Donin AS , Nightingale CM , Owen CG , Rudnicka AR , McNamara MC , Prynne CJ *et al* Ethnic differences in blood lipids and dietary intake between UK children of black African, black Caribbean, South Asian, and white European origin: the Child Heart and Health Study in England (CHASE). Am J Clin Nutr 2010; 92: 776–783. 20739425 10.3945/ajcn.2010.29533 PMC7612313

[14] Vuilleumier J , Keck E . Fluorometric assay of vitamin C in biological materials using a centrifugal analyser with fluorescence attachment. J Micronutrient Anal 1989; 5: 25–34.

[15] Rose D , O'Reilly K , Martin J . The ESRC review of government social classifications. Popul Trends 1997; 49–89. 9368947

[16] Thomas C , Nightingale CM , Donin AS , Rudnicka AR , Owen CG , Sattar N *et al* Socio‐economic position and type 2 diabetes risk factors: patterns in UK children of South Asian, black African‐Caribbean and white European origin. PLoS One 2012; 7: e32619. 22412897 10.1371/journal.pone.0032619 PMC3296720

[17] Cameron ME , van Staveren WA . Manual on methodology for food consumption studies. New York: Oxford Medical Publications, 1988.

[18] Conway JM , Ingwersen LA , Moshfegh AJ . Accuracy of dietary recall using the USDA five‐step multiple‐pass method in men: an observational validation study. J Am Diet Assoc 2004; 104: 595–603. 15054345 10.1016/j.jada.2004.01.007

[19] Donin AS , Nightingale CM , Owen CG , Rudnicka AR , McNamara MC , Prynne CJ *et al* Nutritional composition of the diets of South Asian, black African‐Caribbean and white European children in the UK: the Child Heart and Health Study in England (CHASE). Br J Nutr 2010; 104: 276–285. 20230652 10.1017/S000711451000070X PMC3364483

[20] Fitt E , Cole D , Ziauddeen N , Pell D , Stickley E , Harvey A et al. DINO (Diet In Nutrients Out) ‐ an integrated dietary assessment system. Public Health Nutr 2014: 1–8. 24674815 10.1017/S1368980014000342 PMC4862572

[21] Owen CG , Nightingale CM , Rudnicka AR , Cook DG , Ekelund U , Whincup PH . Ethnic and gender differences in physical activity levels among 9‐10‐year‐old children of white European, South Asian and African‐Caribbean origin: the Child Heart Health Study in England (CHASE Study). Int J Epidemiol 2009; 38: 1082–1093. 19377098 10.1093/ije/dyp176 PMC2720395

[22] Jarvis MJ , Fidler J , Mindell J , Feyerabend C , West R . Assessing smoking status in children, adolescents and adults: cotinine cut‐points revisited. Addiction 2008; 103: 1553–1561. 18783507 10.1111/j.1360-0443.2008.02297.x

[23] Department of Health .National Diet and Nutrition Survey. Headline results from Years 1, 2 and 3 (combined) of the Rolling Programme (2008/2009 ‐ 2010/11). 2012 Department of Health (England).

[24] Song Y , Xu Q , Park Y , Hollenbeck A , Schatzkin A , Chen H . Multivitamins, individual vitamin and mineral supplements, and risk of diabetes among older U.S. adults. Diabetes Care 2011; 34: 108–114. 20978095 10.2337/dc10-1260 PMC3005464

[25] Davie SJ , Gould BJ , Yudkin JS . Effect of vitamin C on glycosylation of proteins. Diabetes 1992; 41: 167–173. 1733805 10.2337/diab.41.2.167

[26] Cutfield WS , Jefferies CA , Jackson WE , Robinson EM , Hofman PL . Evaluation of HOMA and QUICKI as measures of insulin sensitivity in prepubertal children. Pediatr Diabetes 2003; 4: 119–125. 14655269 10.1034/j.1399-5448.2003.t01-1-00022.x

[27] Gregory L , Lowe S . National Diet and Nutrition Survey: young people aged 4 to 18 years. London: The Stationary Office, 2000.

[28] Bates CJ , Thurnham DI . Biochemical markers of nutrient intake In: Margetts B, Nelson M eds. Design concepts in nutritional epidemiology – second edition. Oxford: Oxford University Press, 1997: 209–211.

[29] Schectman G , Byrd JC , Gruchow HW . The influence of smoking on vitamin C status in adults. Am J Public Health 1989; 79: 158–162. 2913833 10.2105/ajph.79.2.158 PMC1349925

[30] Lawlor DA , Davey SG , Kundu D , Bruckdorfer KR , Ebrahim S . Those confounded vitamins: what can we learn from the differences between observational versus randomised trial evidence? Lancet 2004; 363: 1724–1727. 15158637 10.1016/S0140-6736(04)16260-0

[31] Will JC , Byers T . Does diabetes mellitus increase the requirement for vitamin C? Nutr Rev 1996; 54: 193–202. 8918139 10.1111/j.1753-4887.1996.tb03932.x

[32] Will JC , Ford ES , Bowman BA . Serum vitamin C concentrations and diabetes: findings from the Third National Health and Nutrition Examination Survey, 1988‐1994. Am J Clin Nutr 1999; 70: 49–52. 10393138 10.1093/ajcn/70.1.49

