# Supplementary material for: Fruit, vegetable and vitamin C intakes and plasma vitamin C: cross‐sectional associations with insulin resistance and glycaemia in 9–10 year‐old children
Source: Diabet Med. 2015 Nov 23;33(3):307–15. doi: 10.1111/dme.13006 (PMC4832256; doi:10.1111/dme.13006)
Supplement: Supplementary file 1 — Table S1 Blood markers, physical measurements, dietary intake and demographic information by gender Table S2 Correlations between fruit, vegetable, dietary vitamin C intakes and plasma vitamin C Table S3 Percentage and absolute differences in Type 2 diabetes risk markers for specific increases in plasma vitamin C concentrations Table S4 Population characteristics by quartiles of plasma vitamin C Table S5 Percentage differences in Type 2 diabetes risk markers per interquartile range increase in circulating plasma vitamin C concentrations: sensitivity analyses excluding children with high cotinine levels or who reported taking dietary supplements [file DME-33-307-s001.docx]

**Supplementary table S1:** Blood markers, physical measurements, dietary intake and demographic information by gender

† Geometric means and interquartile ranges (IQR) are given for log transformed variables. Means and geometric means, standard deviations (SD) and IQR are based on raw data, p values are adjusted for age, sex, month school as a random effect. ‡ p values for frequencies are derived from chi2 tests.

**Supplementary Table S2:** Correlations between fruit, vegetable, dietary vitamin C intakes and plasma vitamin C

| **All children (n=2,025)** | Fruit intake (grams) | Vegetable intake (grams) | Dietary vitamin C (mg) |
| --- | --- | --- | --- |
| Plasma vitamin C (µmol/L) | 0.11 * | 0.03 | 0.26 * |
| Dietary vitamin C (mg) | 0.32 * | 0.17 * |  |
| **Children who take supplements (n=337)** |  |  |  |
| Plasma vitamin C (µmol/L) | 0.07 | 0.04 | 0.22 * |
| Dietary vitamin C (mg) | 0.25 * | 0.12 * |  |
| **Children who do not take supplements (n=1688)** |  |  |  |
| Plasma vitamin C (µmol/L) | 0.11 * | 0.03 | 0.26 * |
| Dietary vitamin C (mg) | 0.34 * | 0.18 * |  |

* Significant at p<0.05 level

**Supplementary Table S3**: Percentage and absolute differences in type 2 diabetes risk markers for specific increases in plasma vitamin C concentrations

†Absolute differences are calculated in relation to the geometric mean values

IQR = interquartile range

**Supplementary Table S4:** Population characteristics by quartiles of plasma vitamin C

*** Linear regression model.

**†** Kruskal-Wallis tests for trend.

**Supplementary Table S5:** Percentage differences in type 2 diabetes risk markers per interquartile range increase in circulating plasma vitamin C concentrations; sensitivity analyses excluding children with high cotinine levels or who reported taking dietary supplements

Percentage differences are adjusted for age (quartiles), sex, month, total energy intake, adiposity (fat mass index), ethnicity and school (random effect). One IQR increase represents 30.9 µmol/L of plasma vitamin C concentrations.

**Supplementary Figure 1:** Fasting insulin by mean plasma vitamin C (µmol/L) in fourths

Geometric means are adjusted for sex, age (quartiles), ethnic group, month and school (random effect)
