# Supplementary figures and images for: Fruit, vegetable and vitamin C intakes and plasma vitamin C: cross‐sectional associations with insulin resistance and glycaemia in 9–10 year‐old children
Source: Diabet Med. 2015 Nov 23;33(3):307–15. doi: 10.1111/dme.13006 (PMC4832256; doi:10.1111/dme.13006)

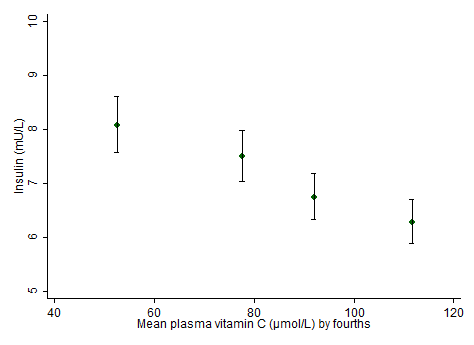

Supplement: Supplementary file 2 — Figure S1 Fasting insulin by mean plasma vitamin C (μmol/l) in fourths. [file DME-33-307-s002.tif]
